# Extract from Prof. Knowles’s Introductory

**Published:** 1855-01

**Authors:** Freeman Knowles

**Affiliations:** Prof. Theory and Practice of Medicine in Iowa Medical Department


### IOWA MEDICAL JOURNAL.

VOL. II. KEOKUK, IOWA, DEC. & JAN., 1854-5. NO. 2.

### ORIGINAL COMMUNICATIONS.

### THE DUTIES OF THE MEDICAL STUDENT.

*Extract from the recent Introductory Address.*

BY FREEMAN KNOWLES, M. D.,

Prof. Theory and Practice of Medicine in Iowa Medical Department.

* * * * Let thoughts of honorable ambition ever fill our minds, and the loftiest eminence of scientific and professional character guide us, as the beacon star in all our efforts. Let us toil for it, and live for it, and the day will come in looking back over the journey of life, when we shall be surprised at the amount of labor performed, the obstacles overcome, and the good attained.

There is hardly any limit to the achievements of energy, combined with determined perseverance. Like the faith spoken of in the scriptures, it removes mountains, and casts them into the sea. It made Demosthenes the prince of Grecian orators.—It enabled Julius Cæsar, although crowned at the early age of eighteen Priest to Jupiter, to win the brightest laurels on the fields of Mars. It enabled Cicero, by fixing his eye upon his great Master, to achieve the reputation that placed him side by side before the world with his Athenian predecessor, and made him the first of Roman Orators. It has made the brightest ornaments in *our* profession, that the world has ever produced. Germany, that land of scholars, folios, lexicons, subtelties and metaphysics, is indebted for its literary character, more to industry and determined perseverance than to any hereditary aptness in the German mind. Sir Isaac Newton, after transcribing with his own hand his famous work on chronology no less than thirteen times, remarks if he has attained any distinction over his fellow men, it has been more from industry and patient thought, than from any mental superiority.—To this untiring energy and determined perseverance, he world has been indebted for all the improvements that have benefited mankind, and shed immortal honors upon their inventors.

It is the possession of these qualities that constitutes the Philosopher's Stone, that has replaced ignorance with knowledge, and dullness with activity; that has changed the old Broadhorn and Keelboat on our rivers, to the magnificent Steamer, the old rickety stage coach, to the snorting iron horse, and the space-annihilating rail-car. This has harnessed the lightning, and compelled it to do our bidding as a carrier boy; has disarmed the portentous thunder-cloud of its forked terrors, and imprisoned the struggling steam, compelling it to toil as a slave for us, to furnish the motive that drives our giant steamers across the pathless ocean, and speeds the flying locomotive along its iron course.

To this same undying energy has the world been indebted for the annihilation of that dreadful scourge of humanity, which annually slew its tens of thousands of victims, the Small Pox, through the agency of vaccination. It has continually been making improvements and discoveries, that annually relieve a large amount of suffering, has lessened nearly two-thirds the period of treatment in some of our miasmatic diseases, and diminished their mortality in almost the same ratio. It has enabled the Surgeon to overcome difficulties in his art, and to treat successfully a great variety of diseases and injuries, that a few years since were considered incurable, thus rendering the surgeon more and more a blessing to mankind.

How important then, that the medical student should be furnished with a well cultivated mind, an energy of character and an indomitable will that knows no such word as failure. With students of that character, the difficulties of professional and scientific studies will flee as the tiny crystals of frost before the rising glories of the morning sun.

But alas for that student (and we fondly hope that we have none such here) who all the time doubts his success. He is very much like a general advancing into an unknown country, who makes more provision for achieving a good retreat, than for overcoming the enemy, should he meet him; continually warning his men of the dangers ahead, and of the importance of being always ready to run away rapidly in case of danger. Such students and such an army are more than half conquered, before the conflict begins. Without complete reformation, such a student may fag on at the foot of his class, but he will always have a character in the profession for stupidity. To such students I would say in all kindness, you have mistaken your vocation and had better turn your attention to some other employment better suited to your habits and capacity, for the profession and the public have a right to expect that he who enters the portals of the temple of medicine shall be no laggart, but earnestly devote whatever of talent and ability God has given him, to the best interests of the science and of his race.

There is however in some minds a great distrust of medical science and medical men, from the fact that discrepancies of opinion exist among the latter, and this is taken as conclusive evidence that there is no certainty or even safety among them, or in any thing they may prescribe. Such persons do not consider that perfection is not the lot of humanity, and that every thing around us partakes of this imperfection; even our nearest friends and most sacred relations participate in this same fundamental defect.

Suppose we were to judge of Law and Divinity by the same rule, how would these professions stand? for they are all based and find their need of activity and usefulness in this very imperfection, so much found fault with in Medicine. Theology is based upon the spiritual imperfections of our race, for if man perfectly comprehended and obeyed all the requirements of his spiritual being, there would be no need of a clergy: they that are whole need not a physician, but they that are sick, is the language of holy writ. We may say the same of the profession of law; it is based upon the social imperfections of our human nature, upon the rivalry of human interests and the zeal of human passions, and hence in civilized life, some means became necessary to settle these conflicting interests, and of this necessity courts of law were born, and have been continually verging towards maturity and perfection. But no one will say that courts of law and equity are useless appendages of society because they are imperfect. When we consider the immense advance which courts have made since the days of Howard, when corruption stalked abroad at noon day, and justice was too often measured by the size of the bribe the parties were enabled to offer, we must be perfectly satisfied of its onward march towards truth and the best civil interests of mankind. So of the profession of medicine; as luxury and a general departure from the primitive habits of our race, began to make their inroads upon the human constitution, it became necessary to find some means to repair the consequences of the transgression of the laws of physical life. Out of this necessity, medical science was born, and amid its thousand difficulties, it has steadily progressed, stemming, as best it might, the torrent of disease and death which vice and luxury have so fearfully accumulated upon it.

How stands Theology relative to varieties of opinion, and this too in christian lands, which of all others we should suppose would boast of their uniformity of faith and practice, founded as all opinions are, upon the infallible and inspired record of the Most High, a volume open to all and free to be judged by all: and yet we find an almost endless variety of opinions. It is said the world has more than five hundred sects, from the most ultra follower of Calvin, to the most wayward and liberal christian speculator. Each and all maintain, with the most untiring zeal, the truth of their several opinions. Perhaps more intellectual labor has been expended, more suffering and torture inflicted, and more blood and carnage exhibited before high heaven, in the vain attempt to make men believe alike, than upon any one subject known in the history of our race. The rack, the dungeon, the stake with fire and faggot, the inquisition with its secret terrors and the guillotine are familiar instruments which have been most fearfully used to promote religious uniformity.

Now where at least in christian lands, all appeal to the same record for their faith and still entertain such endless variety of theological opinions, is it any marvel that men, investigating a living, thinking, intelligent being, with all his idiosyncracies, sympathetic relations, and peculiarities of constitution, with the ever changing character of his diseases as dependent upon season, climate, and miasmatic causes, is it any marvel, I ask, that differences of opinion should exist, at least when, in our investigations of life, its causes and phenomena, we must examine the dead subject, for whenever we invade her sanctuary to scrutinize her mysteries, the coy priestess has fled, and we can only examine the wonderful structure she so recently inhabited.

It is true we have had our forming and transition period, in which hypotheses luxuriated, and imagination wove her dreams of gorgeous imagery, but the cycle of their reign has departed, and the Goddess of Medical Science now admits her votaries to her shrine, only by the slow and patient steps of induction, and any article entering her therapeutia must pass the ordeal of scientific analysis and rigorous experiment. Such being the fact, we may challenge the world to produce a body of men, of any thing like their numbers, with better cultivated minds, embracing a wider range of scientific attainment, or any profession, which by patient toil and profound research, is in possession of more substantial material on which to rest their claims to the consideration of mankind, than the profession of medicine; and we may add, the future permanence and well being, among the literary and scientific associations of the world.

But perfection in any thing is not to be looked for in this our imperfect world. Why then must the profession of Medicine be branded with uncertainty and doubt? Why must the world pass by the other great professions, to slander those who in the hour of distress, are looked upon as angels of mercy? Is it not because we cannot *always* save? If such is the case, all should remember that it is appointed unto man once to die, and no skill of the profession can thwart the will of the Almighty.

But, gentlemen, there is another point to which I wish to call your attention, as exercising a very important bearing upon the success of the Physician. I mean the constant tendency of our race to transgress all the laws of life and health. The human mind has been tortured from time immemorial in devising ways and schemes of physical debasement, till sound health is nearly unknown or known only as the exception, not as the rule. Now if we lived to nature, there would be no good reason why our physical constitution might not be as sound as that of the lower animals.—But so long as we pamper the appetite and gorge the stomach with indigestible compounds, and inflame the blood with narcotic stimulants, we may expect to see ruined constitutions and a low, depraved condition of health. All the skill and ability that the world can command, will not suffice to stem the torrent of disease, degradation, and death, generated by highly seasoned viands and the thousand forms of Alcoholic poison, administered under the delusive names of cordials, wines, brandy, gin, and the whole range of still-house slops, from the best Albany Ale, the united product of dead animals, cocculus indicus, and other deleterious compounds, down thro'

the whole train to Lager Beer, on which so many stupify their brains and ruin their health. Under such a course of training, the constitution is continually becoming undermined, while the organs are stimulated to perform, although very imperfectly, their functions, till nature refuses any longer to work, and the patient thinks he is sick, and malaria, atmospheric vicissitudes, hard work, or some other excuse is relied upon, as the procuring cause of the malady, when in a large majority of cases, the whole difficulty arises from one of two causes ; either from defective organization, inherited from parents or remote ancestors, as the direct consequence of transgressing nature's laws, in the language of holy writ, the sins of the father being visited upon the children to the third and fourth generation ; or it may be to their own neglect of the general laws on which life and health depend. It is to such debased or defective constitutions that a large portion of your investigations will be directed. What marvel then that the physician is not always as successful as could be wished.

Now, while we may safely admit that perfection is not to be found in *our* profession, so we claim that quite as much uncertainty and doubt hangs over a suit at law or any of the peculiar creeds in Theology. While then the law is gradually freeing itself of its musty cobwebs of error, so long sanctified by the lapse of years, and looked up to as venerated authority, so on the other hand, as more light dawns upon the human mind, the true relations of man with man become better understood, new principles are evolved, and justice in the very nature of things, becomes better administered ; for with such a mass of active, intelligent minds acting upon any subject, it would be anomalous if advancement were not visible in its course.

So too with theology. Religion, that golden chain that encircles the universe of intelligent being, how has it been perverted and its fair form calumniated ! Still, with all the errors of earth's mistaken children, it has been the solace and comfort, more or less, to its votaries in the darkest hours of trial and suffering, it has made soft the pillow of the dying ; nay more, it has shone as a pillar of fire to guide a wandering and ignorant world towards its ultimate destiny of virtue and peace, and illumined with transcendant splendor the path from the couch of the dying to the mansions of repose and happiness.

And, as with Law and Divinity, so the Science of Medicine, though cradled in an age of ignorance and superstition, born of necessity, nursed by her mother, true benevolence, has through all her devious wanderings, still approximated to the grand polar star of truth. The errors of each preceding age, have been discarded by a subsequent one, while truth, which is alone immortal, has remained as so much capital stock, to assist in the farther prosecution of the subject. In this way, although false theories and false opinions may become current and rule the passing hour, yet time and experience consign them to their merited oblivion ; while whatever of truth was mixed up with them, becomes sifted out like the gold of California, and remains immortal.

Then gentlemen while you will respect your chosen profession for its antiquity, its erudition, its energy and untiring perseverance, its literature, and its scientific attainments, still you will remember that truth is to be the guiding star of your life. Although your predecessors have achieved many triumphant victories, still the Ultima Thule has not been attained, and it is your duty to add something to the common stock and to make the profession better for your having been associated with it.

When you look around you, and see the teeming earth every where putting forth her green and gorgeous treasures, inviting scrutiny and investigation, the mineral and animal kingdoms with their mysterious agencies so profusely spread out, demanding scientific research, you may in some measure comprehend what lies before you. Who can tell but that in some plant, shrub or tree neglected as useless, may reside a subtle element, that shall stay the destruction which the softened tubercle and scrofulous deposit are making upon the delicate structure of the lungs, and cause the bloom of health to revisit the cheek so recently decked with the hectic flush that bodes only of the tomb ; or some antidote may yet be found, that shall do for the Asiatic Cholera, that dreadful scourge of nations, what vaccination has done for the Small Pox.

The world is full of agencies not yet even dreamed of, that shall prove as potent to the science of Medicine, as electric telegraphs now are to the transmission of news, or the steam engine to perform the ponderous taks which modern civilization has assigned to its iron sinews.

Then, gentlemen, let us devote ourselves to this subject not in a fitful or transitory mood, but with a life-long ardor, and we shall accomplish something worth living for; the science of Medicine may be enriched by our labors, the best interests of our common humanity may be advanced, and our names may descend to future generations as in some degree benefactors of mankind. Remember that this is demanded by your own best interest, by the high expectation of your friends, by the honor of the profession you have espoused, and the vital necessities of a diseased and suffering world.

But gentlemen, there are other duties that pertain to the physician than those of a professional and scientific character. He is to sustain moral and social influences upon the community in which he lives, and as such should be prepared to act his part with intelligence and fidelity in all its social, moral and intellectual relations. * * * Every community has intellectual interests which will more or less demand the care of the educated physician as a prominent member of society. It is a duty society demands of the man, as well as the physician, and should be faithfully performed to the best of his ability.

Ignorance is the parent of degradation, and he who would elevate his race in the scale of moral being, must devise ways and means of educating the masses, for in this way alone can the public mind become disenthralled from false opinions, false systems, and base and immoral practices. These are based upon and find their aliment in gross ignorance. Let the masses be well educated, let a sufficient amount of moral and intellectual aliment be continually furnished them, and the depraved animal appetites will constantly decrease as the moral and intellectual are elevated, till the slave of them shall become emancipated, a renewed moral and intellectual being.

What object more worthy of a high-minded benevolent physician than the elevation of society by moral and inetllectual training?—If all would but devote a portion of their leisure to this subject, in the daily intercourse with their fellow men, an amount of good might be achieved that should tell upon the best interests of our race; and as a small force, acting upon atoms of water, may be communicated from particle to particle in the wide ocean, till it reaches some far distant shore, so such efforts, acting upon the mass of mind, may be felt by generations yet unborn. You are therefore to labor for the well being of society, and in that particular imitate the Friend of sinners, who went about doing good.

Here our profession differs from all others, for in all the higher or more philosophical relations of our profession, our success is against the pecuniary interest of the physician. In our prophylactic and hygenic investigations and regulations, our success is calculated to lessen the amount of disease, and consequently the business of the profession, and yet these very labors are always gratuitous. Who constitute the boards of health of your cities but physicians, and that entirely without emolument, while city attorneys and all other public functionaries are amply remunerated? Who has thrown a wall of defence around the inhabitants of the world to protect them from the ravages of the small pox? Physicians. Who have been the pioneers and supporters in the science of Chemistry?—Who have been the largest contributors to and investigators of geological science but physicians, and have they not done much more than any others in the investigations of Zoology, Botany, Natural History and Vegetable Physiology, besides attending to all that pertains to their immediate profession? Who, let me inquire, has pointed out with such faithful accuracy the pernicious consequences of alcoholic potations upon the human stomach, the brain, the liver, and the circulating fluids of the body, as physicians?

Here, gentlemen, in your intercourse with the world you have an important duty to perform, which if you neglect, you are recreant in your moral duty to society. You must not fail to throw the whole weight of your moral and professional character against the use as a beverage of alcoholic poison. Society demands it of you, first by example that you touch not, taste not, handle not, the seducing poison: this is your first duty, and the second is like unto it, inducing others to do likewise. For when we consider the intimate relations which the family physician sustains to his patrons, and the influence for weal or woe he may exert upon them, how fearful is his responsibility if he be found wanting in this vital quality, of a true friend and safe counsellor! If by his example, he encourage the debasing habit, he may live to feel his own degradation, in the person of a ragged, bloated, imbecile wreck of his former manhood and prospects; or even worse, he may see those who have followed his base practices, the young, the beautiful, the intelligent, filling one after another a drunkard's grave; and it may be the number shall be of his own household, or his own flesh and blood.

Here then, gentlemen, see that you do your duty and your whole duty; see that you are living, acting, aggressive embodiments of temperance and sobriety; demonstrate to the world the impolicy and mal-practice of the use of alcoholic stimulants as a beverage, and deal as sparingly with it in practice as you would with strychnia, arsenic, or hydrocyanic acid. Let me assure you there is no practice so dangerous to the well being of a patient who has ever been an inebriate, as to give him alcoholic stimulants in the low stages of fever; hundreds under such circumstances have risen from the sick bed, only to plunge deeper than ever before into the abyss of drunkenness and degradation, linger on a few years a living monument of disgrace to their friends, themselves and society, to die at last with that terrible disease Delirium Tremens, where the demon of Alcohol revels in satanic glee amid the broken columns, dilapidated arches, and fallen fanes of the soul's temple. Great God, what a wreck! who can contemplate such a scene without emotion, and who can say it had not been far better to let such a patient die, bequeathing the glorious light of his good name to his wife and children, than to live on till the last vestige of reputation has departed amid his bacchanalian revels and drunken orgies.

Ponder these things, gentlemen, and remember that knowing all this, you are in duty bound both to yourself, society, your country, and your God, to use your best influence to dry up this evil which has so long poured disease and death among the sons and daughters of men. * * * Our duty in the premises is plain to a thinking mind; let us make our profession one of progress, in the best sense of the word; seek for truth, wherever it is to be found, whether it be of plebeian or patrician origin; discard error when proved to be such, though hoary with years and stamped with venerable authority. Demonstrate to the world that the regular profession is far better than any of its off-shoots, that we have a larger experience, a more complete materia medica, and that our success in practice is more uniform, based upon better principles, and less hazardous to the lives and constitutions of our patients. Let us keep this object in view, appropriating truth, whether found among the packs and douches of Pressnitz, the antimineral compounds of the Eclectics, or the cayenne pepper, lobelia, and steam of the disciple of Samuel Thompson.—truth, and only truth—discarding the errors of each and all, but relying mainly upon the great store-house of Nature for our materia medica, investigating the laws of life and health with the calmness and breadth of thought of the true philosopher, the patience of an anchorite, and the zeal of a devotee of science. Then we may smile at all attempts to overturn our profession, or even in any degree to permanently mar its prospects, or stay its onward march toward perfection.

